# Association Between Triglyceride–Glucose Index and the Risk of Type 2 Diabetes Mellitus in an Older Chinese Population Aged Over 75 Years

**DOI:** 10.3389/fpubh.2021.796663

**Published:** 2022-03-25

**Authors:** Xiaomin Fu, Hongzhou Liu, Jing Liu, Nan Li, Lele Li, Dianshan Ke, Minyan Liu, Yanhui Lu, Lihua Duan, Linlin Ma, Yanfei Huo, Qinghua Lei, Shuangtong Yan

**Affiliations:** ^1^Department of Endocrinology, The First Medical Center, Chinese PLA General Hospital, Beijing, China; ^2^Department of Endocrinology, First Hospital of Handan City, Handan, China; ^3^Clinics of Cadre, Department of Outpatient, The First Medical Center, Chinese PLA General Hospital, Beijing, China; ^4^Department of Endocrinology, The Second Medical Center and National Clinical Research Center for Geriatric Diseases, Chinese PLA General Hospital, Beijing, China; ^5^Department of Endocrinology, Genetics, Metabolism and Adolescent Medicine, National Center for Children's Health, Beijing Children's Hospital, The Capital Medical University, Beijing, China; ^6^Department of Orthopedics, Fujian Provincial Hospital, Fuzhou, China; ^7^Department of Ultrasound Medicine, Handan Central Hospital, Handan, China; ^8^General Surgery Department 5, Handan Central Hospital, Handan, China; ^9^Physical Examination Center, Handan Central Hospital, Handan, China

**Keywords:** triglyceride–glucose index, type 2 diabetes mellitus, risk, older adults, body mass index

## Abstract

**Background:**

The association between the triglyceride–glucose (TyG) index and type 2 diabetes mellitus (T2DM) in older adults has not been fully understood. This research aims to explore the association between the TyG index and the incidence of T2DM in an older Chinese population aged over 75 years.

**Methods:**

This longitudinal analysis study was performed based on a database from a health check screening program in China. The participants were stratified based on the quintile ranges of the TyG index (Q1 to Q5 groups). T2DM was defined as fasting plasma glucose (FPG) ≥ 7.00 mmol/L and/or self-reported T2DM. The cumulative incidences of T2DM in various quintile groups were estimated by the Kaplan–Meier method. The Cox proportional hazard model was used to examine the independent impact of the TyG index on the risk of T2DM during the follow-up period. Subgroup analysis was performed by gender and BMI to further validate the credibility of the results.

**Results:**

During the follow-up period, a total of 231 new-onset T2DM cases were recorded among the 2,571 individuals aged over 75 years. After adjusting confounding factors, elevated TyG index independently indicated a higher risk of T2DM (HR = 1.89; 95% CI, 1.47–2.44; *p* < 0.01). Higher TyG index quintile groups (Q3 to Q5) also presented with a higher risk of T2DM (hazard ratio (HR) = 1.36, 1.44, and 2.12, respectively) as compared with the lowest quintile group (Q1). Subgroup analysis showed that increased TyG index led to a higher risk of T2DM with HR = 2.35 (95% CI, 1.73–3.19), 1.90 (95% CI, 1.27–2.83), 2.95 (95% CI, 1.94–4.50), and 1.72 (95% CI, 1.25–2.35) in male subgroup, female subgroup, BMI < 24 kg/m^2^ subgroup, and BMI ≥ 24 kg/m^2^ subgroup, respectively.

**Conclusions:**

Triglyceride–glucose index independently correlated with the risk of incident T2DM in Chinese adults aged over 75 years. The TyG index might be useful in monitoring T2DM in the older populations.

## Introduction

Type 2 diabetes mellitus (T2DM) has been considered as a risk equivalent for cardiovascular disease and all-cause mortality, which imposes a remarkable economic burden on patients and societies (1, 2). In recent decades, the prevalence of patients with T2DM has significantly increased, especially in developing countries (3). Chinese adults, especially older adults, have experienced a distinct transition of diet patterns and a tremendous rise in the incidence of obesity, which is also a major trigger for the development of T2DM (4, 5). In line with the statistics by the International Diabetes Federation (6), China has the largest population of patients with T2DM, approaching 114.4 million in 2017. Currently, epidemiological investigation reveals that there exist over 400 million patients with diabetes in the world, and this figure is estimated to increase to 700 million by 2045. T2DM is a leading cause of morbidity and mortality in the geriatric population. Other concomitant diseases in older patients with T2DM, such as renal dysfunction, heart failure, stroke, dementia, muscle loss, cognitive impairment, and osteoporosis, also contribute to the difficulty in T2DM management. However, the specific characteristics of geriatric T2DM have not been given due attention in previous studies. Hence, regular health checks, early identification of T2DM, and early intervention are the major important factors to prevent the development of T2DM and its related complications so as to improve the quality of life of older adults. Besides, surveillance of T2DM needs to be individualized in this age group, keeping in mind the benefit to risk ratio. Thus, a simple and cost-effective predictor is warranted for detecting individuals in the older population with a high risk of T2DM.

The main risk factors of T2DM include an unhealthy diet, obesity, sedentary behaviors, and reduced physical activity (7). The insulin resistance (IR) and dysfunction of islet β-cell are the pivotal pathophysiological pathways of T2DM (8, 9). IR is presented as insulin-dependent organic cells that respond inappropriately to insulin stimulation (10) and may occur as early as ~20 years prior to the definite diagnosis (9, 11). Moreover, the aging-related pathophysiological changes in older adults can lead to enhanced susceptibility of T2DM and carbohydrate intolerance, which are due to the decrease in insulin secretion following glucose load and also the aggravation in IR in tissues (12). A recent research suggested that IR was more significantly related to the onset of diabetes mellitus in the Chinese population than β-cell dysfunction (13). IR is associated with multiple metabolic abnormalities that include dyslipidemia and hyperglycemia (10).

In brief, the evaluation of IR status is necessary to identify high-risk population of T2DM. The typical methods to assess IR, such as hyperglycemic clamp and the homeostasis model assessment of IR (HOMA-IR), are costly and time-consuming for routine medical examination and large-scale epidemiological investigations. However, regular health checkup requires noninvasive and cost-effective tests to identify those with a greater risk of T2DM. Recently, the triglyceride–glucose (TyG) index, calculated by the formula ln(fasting triglycerides(mg/dl)^*^fasting blood glucose (mg/dL)/2) (14), has been found to be associated with several commonly used alternate predictors of IR that include hyperglycemic clamp and HOMA-IR (14–16). It has also been considered as a candidate indicator for defining the status of metabolic health (17). Moreover, recent evidence suggests that the TyG index is highly correlated with arterial stiffness in both healthy individuals and patients with hypertension (18, 19) and could predict the risk of adverse cardiovascular events in the T2DM population (20).

In the consideration of the feature of the TyG index as a credible and surrogate indicator of IR, it might also be a potential predictor of T2DM. Compared with insulin-based indices, the TyG index could be easier to obtain and calculate for clinical investigators, and several epidemiologic studies have indicated that the TyG index is related to the risk of incident T2DM in Asian and European countries (20–29). However, the outcomes were inconclusive and controversial as a result of either the cross-sectional design (30), limited population scale (29), or confined study population such as normal-weight adults (21, 31). Nonetheless, the association between the TyG index and incident T2DM has not been investigated in older adults. Therefore, in our study, data were downloaded freely from a public database based on a population cohort in China, and a longitudinal analysis was performed. We aimed to explore the relationship of the TyG index with the risk of developing incident T2DM in an older Chinese population aged over 75 years.

## Methods

### Research Design

We downloaded the original database sorted by Chen et al. (32) from the DATADRYAD website (www.datadryad.org), where original databases can be freely obtained by others. The original database was designed based on a population cohort from 2010 to 2016 in China with a median follow-up of 3.1 years, and the data were acquired for the public database established by the Rich Healthcare Group (32). The inclusion criteria for the participants in the database were as follows: (1) at least 20 years old; (2) followed up for at least 2 years; and (3) with available data of body mass index (BMI) and fasting plasma glucose (FPG) value. In the study by Chen et al., the population cohort investigation was approved by the Rich Healthcare Group Review Board (32), and thus, ethics approval was not required in this longitudinal analysis. According to the statement in the study by Chen et al. (32), the authors permitted others to perform secondary analysis based on their work non-commercially. Therefore, their database was used in this longitudinal analysis without infringing the authors' rights. Since several previous studies reported that 75 years old is considered a watershed for the significant deterioration of many of the body systems and also the development of diseases such as T2DM, hypertension, and cardiovascular or cerebrovascular diseases, we only included participants over 75 years old to investigate the incident T2DM in an older Chinese population. The following patients were excluded: (1) participants with missing TyG index measurements; (2) patients lost to follow-up; (3) patients with visit intervals <2 years; and (4) patients with extreme BMI values (<15 kg/m^2^ or >55 kg/m^2^) (32). Then, the general information, biochemical test results, and the incidence of T2DM of the participants were acquired. Based on these data, the association between the TyG index and the risk of incident T2DM was investigated.

### Definitions

The endpoint of the study was the diagnosis of T2DM during the follow-up period or during the last visit. The definition of incident T2DM in the research by Chen et al. (32) was FPG ≥ 7.00 mmol/L and/or self-reported T2DM by the patients.

### Data Acquisition

In the database offered by Chen et al. (32), the baseline examinations were performed by using the standardized spreadsheet to acquire the participant's general information and by testing the laboratory indices under the fasting status. The following data were recorded and analyzed: (1) basic information and anthropometric indices that include age, gender, blood pressure, weight (kg), height (m), and BMI [calculated as weight (kg)/height (m2)]; (2) laboratory parameters that include triglyceride, alanine aminotransferase, aspartate aminotransferase, blood urea nitrogen, endogenous creatinine clearance rate, total cholesterol, low-density lipoprotein cholesterol, high-density lipoprotein cholesterol, and FPG; (3) sociodemographic parameters such as smoking history and alcohol intake; and (4) the study endpoint (incident T2DM).

### Statistical Analysis

The missing values of the variables were supplemented using multiple imputations prior to statistical analysis, based on a multiple of replications and a chained equation approach modality in the R language. For quantitative variables, values are shown as mean ± standard deviation, and the differences between groups were compared *via* the one-way analysis of variance (ANOVA) method. For qualitative variables, data are presented as numbers (percentages), and the significance of differences between groups was analyzed through the chi-square test.

To explore the association of different TyG index values with the risk of T2DM onset, patients aged over 75 years were stratified into five groups according to the quintile values of the TyG index of all participants, namely, Q1, Q2, Q3, Q4, and Q5 groups. The independent impact of the TyG index as a continuous variable or as a hierarchical variable (Q1, Q2, Q3, Q4, and Q5 groups) on the risk of incident T2DM was evaluated *via* the Cox proportional hazard model. The crude (univariate) model only included the TyG index. Model I was adjusted for gender, age, and BMI. Model II (fully adjusted model) was adjusted for gender, age, BMI, systolic blood pressure, alanine aminotransferase, and blood urea nitrogen. The *p*-Values of the Cox regression analysis and corresponding hazard ratios with 95% confidence intervals (CIs) were recorded and presented. The log-rank test was performed to compare the T2DM risks among TyG index quintile groups, and the Kaplan–Meier curves for T2DM onset were applied for generating cumulative event rates. Given the potential confounding effects of gender and BMI, the gender-stratified as well as BMI-stratified (cutoff value, 24 kg/m2) multivariate Cox regression proportional hazards models were analyzed, with adjustment for gender, age, BMI, systolic blood pressure, alanine aminotransferase, and blood urea nitrogen. A subgroup analysis was performed by gender and BMI to further validate the results. As is known, a higher BMI is associated with a greater risk of T2DM. According to the criteria of weight for adults issued by the National Health and Family Planning Commission of the People's Republic of China, BMI ≥ 24 kg/m^2^ is considered overweight (33). Therefore, we divided BMI as < 24 kg/m^2^ and ≥ 24 kg/m^2^ in this study based on a Chinese population (33).

Data analysis and plotting were performed using R language (version 4.1.0; R Foundation for Statistical Computing) and SAS version 9.2 (SAS Institute Inc, Cary, NC). The *p*-value < 0.05 from the two-sided hypothesis test was considered statistically significant.

## Results

### Baseline Characteristics and the Incidence of T2DM of the Included Participants

A total of 2,571 participants aged over 75 years were included in this longitudinal analysis. Table 1 shows the baseline characteristics of the included participants stratified by quintile of the TyG index.

**Table 1:** Baseline characteristics of the participants.

**Variable**	**All participants** **(2,571)**	**TyG index**					***P* value**
		**Q1 (514)**	**Q2 (514)**	**Q3 (513)**	**Q4 (515)**	**Q5 (515)**	
Age	79.68 (4.06)	80.15 (4.39)	79.73 (4.17)	79.77 (4.04)	79.39 (3.77)	79.38 (3.85)	0.013
Gender							<0.001
Male	1,634 (63.6)	379 (73.7)	352 (68.5)	314 (61.2)	293 (56.9)	296 (57.5)	
Female	937 (36.4)	135 (26.3)	162 (31.5)	199 (38.8)	222 (43.1)	219 (42.5)	
Bmi	23.96 (3.18)	22.73 (3.06)	23.57 (3.28)	23.89 (3.16)	24.54 (2.97)	25.08 (2.89)	<0.001
Tyg index	8.60 (0.51)	7.90 (0.22)	8.33 (0.08)	8.59 (0.08)	8.86 (0.08)	9.33 (0.26)	<0.001
Systolic blood pressure	130.37 (12.18)	128.73 (13.09)	129.53 (12.34)	130.31 (12.18)	131.46 (11.70)	131.80 (11.31)	<0.001
Diastolic blood pressure	76.29 (8.93)	75.42 (8.66)	75.59 (8.90)	76.15 (9.04)	77.34 (9.26)	76.94 (8.63)	0.001
Fasting blood glucose	5.14 (0.47)	4.98 (0.46)	5.08 (0.44)	5.14 (0.46)	5.22 (0.44)	5.30 (0.47)	<0.001
Total cholesterol	5.01 (0.73)	4.72 (0.70)	4.91 (0.73)	5.08 (0.73)	5.11 (0.68)	5.23 (0.71)	<0.001
Triglyceride	1.51 (0.83)	0.70 (0.15)	1.04 (0.13)	1.33 (0.15)	1.71 (0.21)	2.79 (0.87)	<0.001
High-density lipoprotein	1.38 (0.24)	1.44 (0.24)	1.41 (0.24)	1.40 (0.24)	1.35 (0.23)	1.30 (0.23)	<0.001
Low-density lipoprotein	2.90 (0.54)	2.72 (0.51)	2.87 (0.54)	2.98 (0.55)	2.99 (0.52)	2.94 (0.55)	<0.001
Alanine aminotransferase	17.00 [13.30, 22.00]	15.50 [12.33, 19.50]	16.35 [13.20, 21.37]	16.20 [13.00, 21.50]	18.00 [14.00, 22.35]	18.00 [15.00, 24.30]	<0.001
Aspartate aminotransferase	24.00 [21.00, 28.25]	24.00 [21.00, 28.00]	24.00 [21.00, 28.10]	23.50 [20.70, 28.00]	24.80 [21.00, 28.00]	24.80 [21.50, 29.20]	0.060
Blood urea nitrogen	5.08 (0.92)	5.20 (0.90)	5.06 (0.93)	5.06 (0.91)	5.01 (0.92)	5.08 (0.91)	0.016
Creatinine clearance rate	75.09 (11.89)	76.08 (11.34)	75.32 (11.63)	74.71 (12.15)	73.98 (12.20)	75.37 (12.07)	0.060
Type 2 diabetes mellitus	231 (9.0)	33 (6.4)	27 (5.3)	44 (8.6)	49 (9.5)	78 (15.1)	
Smoking							0.001
Every day	716 (27.8)	176 (34.2)	145 (28.2)	146 (28.5)	123 (23.9)	126 (24.5)	
Occasionally	119 (4.6)	21 (4.1)	34 (6.6)	16 (3.1)	23 (4.5)	25 (4.9)	
None	1,736 (67.5)	317 (61.7)	335 (65.2)	351 (68.4)	369 (71.7)	364 (70.7)	
Drinking							0.185
Every week	40 (1.6)	4 (0.8)	13 (2.5)	10 (1.9)	8 (1.6)	5 (1.0)	
Occasionally	291 (11.3)	67 (13.0)	60 (11.7)	58 (11.3)	46 (8.9)	60 (11.7)	
None	2,240 (87.1)	443 (86.2)	441 (85.8)	445 (86.7)	461 (89.5)	450 (87.4)	

The average age of the study population was 79.68 ± 4.06 years, and 63.6% of them were men.

Participants in the higher quintile groups of TyG index (Q2, Q3, Q4, and Q5) showed higher levels of BMI, systolic blood pressure, diastolic blood pressure, fasting blood glucose, total cholesterol, triglyceride, low-density lipoprotein, and alanine aminotransferase and lower levels of high-density lipoprotein, blood urea nitrogen, creatinine clearance rate, and smoking frequency as compared to Q1 group.

**Figure 1:**
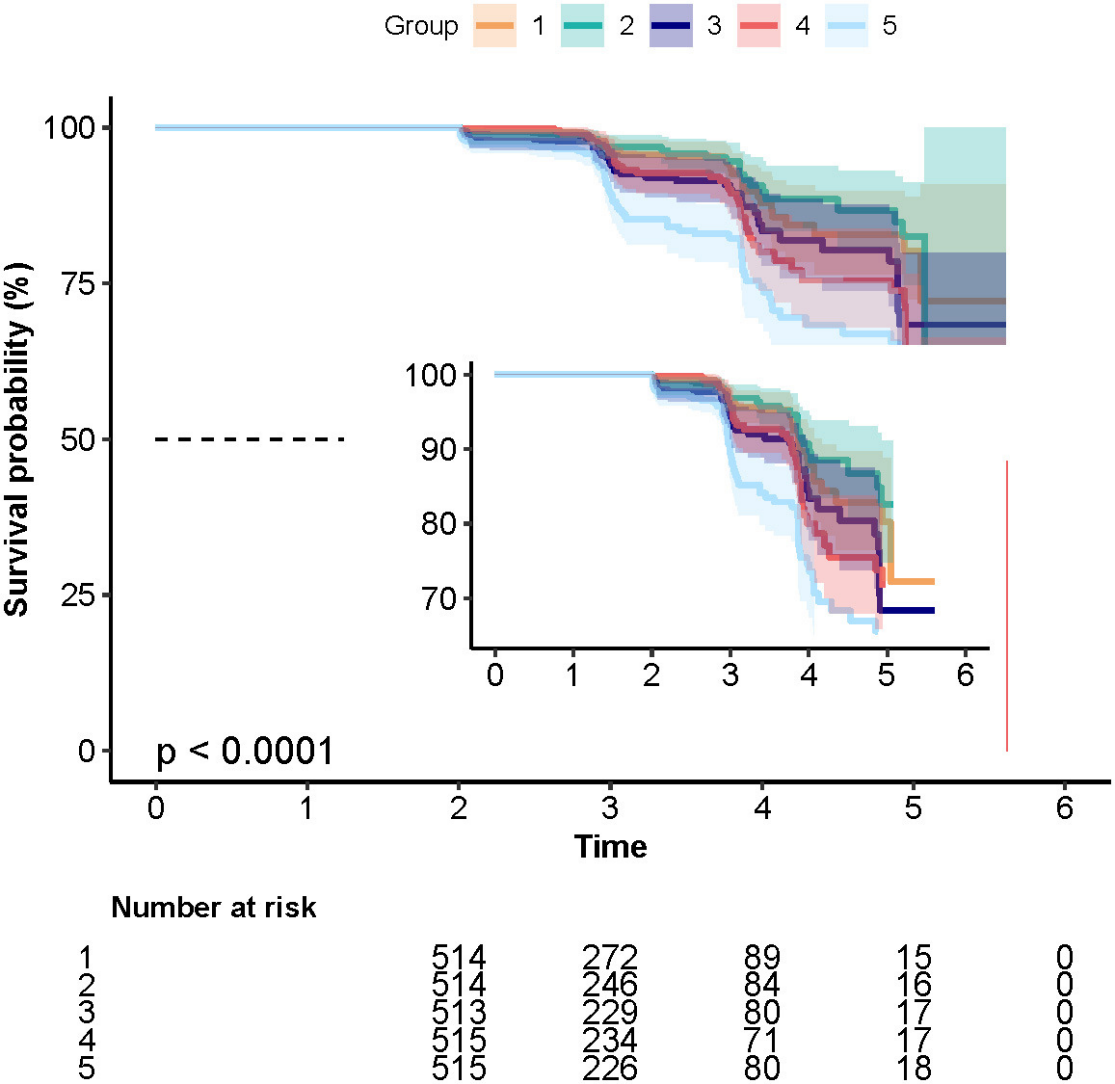
The Kaplan–Meier curves depicting the cumulative incident type 2 diabetes mellitus (T2DM) in different quintile groups (Q1 to Q5) of triglyceride-glucose (TyG) index. Each color of lines indicates a quintile group. The color range indicates the 95% confidence internval (CI) range of cumulative incidence of T2DM at a different follow-up time.

During the follow-up period, 231 cases of T2DM were identified with an incidence of 9%. As shown in Table 1 and Figure 1, a total of 33 (6.4%), 27 (5.3%), 44 (8.6%), 49 (9.5%), and 78 (15.1%) cases of T2DM were observed in Q1, Q2, Q3, Q4, and Q5 groups, respectively.

### Unadjusted and Adjusted Cox Proportional Hazard Models of Incident T2DM

The independent impact of the TyG index on the risk of incident T2DM was evaluated using the unadjusted (univariate) and adjusted (multivariate) Cox proportional hazard models. The variables added to the different adjusted models were selected based on the results of an univariate analysis. The effect sizes of the TyG index, including hazard ratio (HR) and 95% CIs, are depicted in Table 2. Higher TyG index increased the hazard of T2DM development (HR = 1.89; 95% CI, 1.47–2.44) after adjusting for gender, age, BMI, systolic blood pressure, alanine aminotransferase, and blood urea nitrogen. As for sensitivity analysis, the quintile of the TyG index was used as a categorical variable, and the hazard ratio as well as the *p*-value for the trend of T2DM risk were calculated. The hazard ratio of T2DM in the whole adjusted model was 1.97 (95% CI, 1.45–2.66; *p* < 0.01).

**Table 2:** Multivariate analysis for the relationship between TyG index and incident T2DM.

	**Hazard ratio, 95% CI and** ***P*** **value**		
	**Crude**	**Model I**	**Model II**
TyG index	2.21 (1.74, 2.82) < 0.01	1.98 (1.54, 2.55) < 0.01	1.89 (1.47, 2.44) < 0.01
TyG index quintiles			
Q1	1	1	1
Q2	0.88 (0.53, 1.46) 0.62	0.84 (0.51, 1.41) 0.52	0.83 (0.50, 1.39) 0.48
Q3	1.52 (0.97, 2.39) 0.07	1.39 (0.88, 2.20) 0.15	1.36 (0.86, 2.14) 0.19
Q4	1.69 (1.08, 2.62) 0.02	1.49 (0.95, 2.34) 0.08	1.44 (0.92, 2.27) 0.11
Q5	2.67 (1.77, 4.01) < 0.01	2.24 (1.47, 3.42) < 0.01	2.12 (1.39, 3.24) < 0.01
*P* for trend	2.35 (1.76, 3.14) < 0.01	2.05 (1.52, 2.77) < 0.01	1.97 (1.45, 2.66) < 0.01

### Subgroup Analysis

Table 3 shows the subgroup analysis for the association between the TyG index and T2DM development after adjusting for gender, age, BMI, systolic blood pressure, alanine aminotransferase, and blood urea nitrogen. Subgroups were stratified based on gender (male and female) and BMI (< 24 kg/m^2^ and ≥ 24 kg/m^2^). The results suggested that an elevated risk of incident T2DM stably existed in the different subgroups in Q3, Q4, and Q5 groups as compared to the Q1 group (HR > 1 for all), and there were statistical significances for the Q5 group vs. Q1 group (*p* < 0.05 in all subgroups). When analyzed as a continuous variable, the elevation of TyG index resulted in an increased risk of incident T2DM by 2.35-folds (95% CI, 1.73–3.19; *p* < 0.01), 1.90-folds (95% CI, 1.27–2.83; *p* < 0.01), 2.95 (95% CI, 1.94–4.50; *p* < 0.001), and 1.72-folds (95% CI, 1.25–2.35; *p* < 0.01) in the male subgroup, the female subgroup, BMI < 24 kg/m^2^ subgroup, and BMI ≥ 24 kg/m^2^ subgroup, respectively.

**Table 3:** Subgroup analysis.

**Confounding factor** **category**	**Serum TyG index quintiles**					**HR for TG/HDL-C as continuous variable**	***P* for trend**	***P* for interaction**
	**Q1**	**Q2**	**Q3**	**Q4**	**Q5**			
Gender								0.39
Male	1	0.70 (0.37, 1.30) 0.26	1.10 (0.61, 1.96) 0.76	1.53 (0.90, 2.61) 0.12	2.69 (1.66, 4.36) < 0.01	2.35 (1.73, 3.19) < 0.01	2.57 (1.78, 3.72) < 0.01	
Female	1	1.47 (0.59, 3.66) 0.41	1.08 (1.09, 5.48) 0.03	1.40 (0.91, 4.73) 0.08	1.26 (1.24, 5.99) 0.01	1.90 (1.27, 2.83) < 0.01	1.88 (1.18, 3.02) < 0.01	
BMI								0.045
< 24	1	0.66 (0.31, 1.42) 0.289	1.30 (0.66, 2.57) 0.44	1.44 (0.72, 2.88) 0.30	3.83 (2.14, 6.87) < 0.01	2.95 (1.94, 4.50) < 0.001	3.25 (2.01, 5.25) < 0.01	
≥24	1	1.03 (0.50, 2.08) 0.95	1.50 (0.79, 2.83) 0.22	1.55 (0.83, 2.88) 0.17	1.90 (1.05, 3.45) 0.03	1.72 (1.25, 2.35) < 0.01	1.68 (1.15, 2.45) < 0.01	

## Discussion

This longitudinal analysis study showed that increased TyG index was independently associated with a higher risk of incident T2DM in an apparently healthy older population aged over 75 years in China (HR = 1.89; 95% CI, 1.47–2.44; *p* < 0.01). As compared to the individuals with the lowest quintile of the TyG index, participants with the top quintile of the TyG index presented with a 2.1-fold higher risk of T2DM onset (Q5 vs. Q1: adjusted HR = 2.12; 95% CI, 1.39–3.24; *p* < 0.01). Besides, the results of subgroup analysis demonstrated that this association existed in spite of difference in gender (male or female) or BMI (< 24 kg/m^2^ or ≥ 24 kg/m^2^), which indicates that the conclusions were robust and the TyG index was applicable for the older subjects. Additionally, stronger correlations were discovered in male individuals and in individuals with BMI < 24 kg/m^2^.

The TyG index is a product derived from triglyceride and fasting blood glucose and has been found to be a potential biomarker for IR in previous epidemiological investigations (14, 34–36). Guerrero-Romero et al. found not only a considerably high sensitivity (96.5%) but alsoa satisfactory specificity (85.0%) of the TyG index for detecting IR in a Mexican population (15). The TyG index also had better performance than HOMA in a Brazilian investigation by Vasques et al. (37). In accordance with the results of this study, some previous investigations also suggested that an increased TyG index might be associated with a higher risk of future T2DM onset in multiple regions in Asia and Europe (22, 28, 29, 38). A previous Chinese cohort study also found the consistent results in individuals with normal BMI (21). Thus, the relationship between the TyG index and T2DM risk among older Chinese individuals has been rarely investigated in previous studies. Since this study was carried out based on a large cohort of 2,571 apparently healthy old adults aged over 75 years with all range of BMI values in China, it is applicable to a considerably wide range of the older population and provides a reliable basis for further clinical promotion and practice.

Type 2 diabetes mellitus is characterized by islet β-cell dysfunction and IR (39). Notably, the TyG index might also be a predictor for the susceptibility of β-cells to the cytotoxic effect of glucose and lipid. Islet β-cell is known to be vulnerable to oxidative stress due to limited antioxidant enzyme ability, and oxidative stress has been found to be involved in the pathogenesis and progression of T2DM (40, 41). Moreover, emerging evidence has found that certain antioxidant supplementation could modulate lipid metabolism and enhance insulin sensitivity (42–44). Increased blood glucose contents also induce reactive oxygen species production in islet β-cells, which in turn leads to glucose toxicity and dysregulated function of β-cells, thus promoting IR and T2DM (40, 41). Besides, the dysfunction of pancreatic β-cells can also be induced by long-term exposure to triglyceride, which might be caused by continuously elevated free fatty acid levels (45, 46). Therefore, to a certain extent, the TyG index reflects the transformation of physiological modulation and metabolic regulation of the body and is closely associated with the probability of the onset and progression of T2DM.

To further explore the substantial correlations between independent and dependent variables in the onset of T2DM, we performed subgroup analysis and explored potential interactions. In this study, gender and BMI were taken as grouped variables, and more significant correlations were found in male individuals and those with BMI ≥ 24 kg/m^2^. This hazard ratio was relatively larger in male participants, which was inconsistent with the cohort investigation by Zhang et al. (21). This discordance might be partially due to the different age distributions in our study population, which were all aged > 75 years. With the aging process in older adults, the lipid deposition in hepatocytes in men might have different patterns as compared to women (47, 48). On the other hand, it is well known that obese individuals are heterogenous from lean ones and are usually considered to be more susceptible to T2DM. However, in the subgroups according to the cutoff value of BMI, TyG index showed a significantly positive correlation with the risk of future T2DM, and lean individuals presented with a higher hazard of future T2DM risk with increased TyG index (BMI < 24 kg/m^2^, HR = 2.95, *p* < 0.001; BMI ≥ 24 kg/m^2^, HR = 1.72, *p* < 0.01). A possible reason for this inconsistency from expectation was that the mechanism of the TyG index promoting T2DM in various BMI populations might be diverse. The increased risk of T2DM in obese populations with high TyG index might be mainly caused by aggravated IR, whereas in the lean population, the dysfunction of β-cells induced by glycotoxicity and lipotoxicity should be attributed to. Taken together, according to the results of the subgroup analysis, the TyG index showed a higher sensitivity for estimating the risk of T2DM in the male population and those with normal BMI. Thus, it might be a useful index for monitoring the risk of T2DM onset, especially in older male populations without obesity. For instance, the TyG index could be calculated and provided for individuals at regular health checks with the corresponding explanations such as “low/moderate/high risk of T2DM” and suggestions such as “re-check within 3/6/12 months at endocrine clinic.”

The present research has several strengths. First, this investigation is performed based on a large cohort study with age >75 years, and thus, the results are specifically applicable and dependable for the older population. The other similar clinical observations either had a relatively small population or the age of the sample tended to be diverse. Second, we have taken the TyG index as both a ranked categorical variable and as a continuous variable, and sensitivity calculation and trend examination were both performed to enhance the credibility of the conclusion. There are also a few limitations to this study. First, T2DM was diagnosed based on fasting blood glucose without the data of glycosylated hemoglobin (HbA1c) and oral glucose tolerance test, which might result in the underestimated incidence of T2DM. Second, due to the lack of data on insulin levels and HbA1c, the comparison of the TyG index and HOMA-IR or HbA1c in predicting T2DM risk is not available in this study. Finally, since this cohort research was conducted in China, the findings of this study need to be validated in other races and a worldwide range of populations.

## Conclusion

The present investigation suggests that increased TyG index is independently associated with a higher risk of T2DM onset in the older Chinese population aged over 75 years. Moreover, the results in this study extend our knowledge that the TyG index appears to be more sensitive for evaluating the risk of T2DM in men as well as in lean individuals. The TyG index might thus be a useful tool for detecting individuals at A high risk of developing T2DM, especially in men without obesity.

## Data Availability Statement

The original contributions presented in the study are included in the article/supplementary material, further inquiries can be directed to the corresponding authors.

## Ethics Statement

Ethical approval was not provided for this study on human participants because the ethics approval was obtained in the previous research by Chen et al. (32) and was no longer needed for the current study. The patients/participants provided their written informed consent to participate in this study.

## Author Contributions

QL and SY contributed to the conception and design of the research. YL, LD, LM, and YH acquired the data from the database. LL, DK, NL, and ML performed the statistical analysis. XF, HL, and JL drafted the manuscript. All authors contributed to the article and approved the submitted version.

## Conflict of Interest

The authors declare that the research was conducted in the absence of any commercial or financial relationships that could be construed as a potential conflict of interest.

## Publisher's Note

All claims expressed in this article are solely those of the authors and do not necessarily represent those of their affiliated organizations, or those of the publisher, the editors and the reviewers. Any product that may be evaluated in this article, or claim that may be made by its manufacturer, is not guaranteed or endorsed by the publisher.
